# Inattention and hyperactivity in children and adolescents with repaired D-transposition of the great arteries: Prevalence, perioperative risk factors, and clinical outcomes

**DOI:** 10.3389/fcvm.2022.937311

**Published:** 2022-09-20

**Authors:** Hongtong Chen, Yichen Yan, Cong Li, Xiangyu Zheng, Guanghai Wang, Zhijuan Jin, Guocheng Shi, Xiaomin He, Xiaoping Tong, Huiwen Chen, Zhongqun Zhu

**Affiliations:** ^1^Department of Cardiothoracic Surgery, Heart Center, Shanghai Children’s Medical Center, Shanghai Jiao Tong University School of Medicine, Shanghai, China; ^2^Center for Brain Science, Shanghai Children’s Medical Center, Shanghai Jiao Tong University School of Medicine, Shanghai, China; ^3^Peking University Sixth Hospital, Peking University Institute of Mental Health, Beijing, China; ^4^Department of Developmental and Behavioral Pediatrics, Shanghai Children’s Medical Center, Shanghai Jiao Tong University School of Medicine, Shanghai, China; ^5^Department of Anatomy and Physiology, Shanghai Jiao Tong University School of Medicine, Shanghai, China

**Keywords:** transposition of the great arteries, attention-deficit/hyperactivity disorder, attention deficit symptom, prevalence, risk factors

## Abstract

**Objective:**

The present study objectives were to determine the prevalence of attention-deficit/hyperactivity disorder symptoms (ADHD-like symptoms) in children and adolescent with d-transposition of great artery (D-TGA) after arterial switch operation (ASO) and examine associated risk factors and adverse personal, family dysfunctions.

**Methods:**

This cohort study included 103 patients with D-TGA who underwent ASO in early infancy at Shanghai Children’s Medical Center between 2011 and 2016 and then follow-up. Data analysis was conducted from September 2020 to April 2022. A standardized Swanson, Nolan, and Pelham IV (SNAP-IV) questionnaire is used to evaluate inattention and hyperactivity symptoms. Demographic, preoperative, intraoperative, and postoperative factor were collected. Univariate and multivariable regression analyses were performed with odds ratios (OR) and 95% confidence intervals (CIs).

**Results:**

Prevalence of ADHD-like symptoms was 27.18% (28/103). Attention-deficit (18/28, 64.29%) symptom was the predominant subphenotype. After underwent TGA surgery, 39% of patients with ADHD-like symptoms receive remedial special academic services. There is none had repeated grade. Univariate analysis showed that, positive inotropic drug score (*P* = 0.03) and delayed sternal closure (*P* = 0.02) were risk factors of ADHD-like symptoms; increased preoperative oxygen saturation (SpO_2_) (*P* = 0.01) and surgical height (*P* = 0.01) and TGA subtype (VSD) (*P* = 0.02) were protective factor of ADHD-like symptoms. Multivariable analysis showed that delayed sternal closure (DSC) (OR, 1.50; 95% CI, 1.02–2.18) is a risk factor for the occurrence of ADHD-like symptom while increased preoperative oxygen saturation [odds ratio (OR), 0.95; 95% confidence interval (CI), 0.92–0.99] is a protective factor of ADHD-like symptom.

**Conclusion:**

The children and adolescents with D-TGA after ASO were at high risk of ADHD-like symptoms. Preoperative hypoxic status and postoperative DSC became predominant risk factors. Modification of the risk factors may be helpful to relieve ADHD-like symptoms for these patients.

## Introduction

Congenital heart disease (CHD) is the most common congenital malformation, accounting for about 2–3% of population. D-transposition of the Great Arteries (TGA) is a misalignment of the main ventricular arteries, showing that the main artery starts from the right ventricle while the pulmonary artery starts from the left ventricle. This anatomical anomaly makes the systemic and pulmonary circulation become two sets of parallel cycles. Shunt which depending on the levels of atria, ventricles, or aorta allows children to survive with mixed blood between the two circulations. TGA accounted for about 3% of all CHD and nearly 20% of all cyanosis CHD. Without timely treatment, the mortality rate of d-transposition of great artery (D-TGA) children within 1 year is 90% (1). D-TGA is also one of the most complex congenital heart diseases (CHD) in the neonatal period. Arterial switch operation (ASO) is the gold-standard surgical approach for D-TGA, after surgery can reduce the mortality to 5–10% (2). With advances in surgical techniques, perfusion protocols, and perioperative intensive care, the surgical mortality for this lesion is very low, now less than 5% in many medical centers (3–5). However, the morbidity of neurodevelopmental disorders including academic achievement, memory, executive functions, visual-spatial skills, attention, and social cognition was common at school age, 65% children and adolescents received remedial academic or behavioral services (4). There are many neurodevelopmental disorders in TGA, which may be caused by hemodynamic blood flow during the fetal period, surgical factors during the operation and postoperative socio-economic status. The mechanism is unclear now. Some studies have reported that attention deficit hyperactivity disorder (ADHD) symptoms accounts for 20% (6) in TGA patients. However, the prevalence, risk factors and clinical outcomes of ADHD-like symptoms in TGA have not been reported. Therefore, neurodevelopmental assessment, follow-up, and improving long-term outcomes after ASO have become increasing important (3–5).

ADHD is a common neurobehavioral and psychiatric disorder characterized by inattention and/or hyperactivity and impulsivity with an estimated prevalence rate around 5% in children, and frequently persists into adult, estimated to be around 2.5% (7, 8). It causes serious personal dysfunctions including impaired academic, occupational, and social functioning; increased rates of substantial comorbidity including substance use, depression, anxiety, and accidents; and increased costs to the family, school and society (7, 8). Previous studies have suggested that attention and hyperactive symptoms are frequent in children with CHD (9, 10) and the risk of ADHD is 3–4 times higher in patients with complex CHD than in the general population (11). However, the occurrence and clinical characteristics of ADHD, and perioperative risk factors in D-TGA patients after ASO have not been extensively studied. The primary purpose of our study was to analyze the prevalence of ADHD-like symptoms after TGA surgery. The secondary purpose was to identify perioperative risk factors for adverse outcomes. Therefore, our hypothesis is that the prevalence of ADHD-like symptoms is high after surgery of TGA.

## Materials and methods

### Study participants

In this cohort study, consecutive patients (*n* = 341) mainly diagnosed with transposition of TGA were retrospectively identified from the hospital database in Shanghai Children’s Medical Center between 2011 and 2016 and follow-up. Data analysis was conducted from September 2020 to April 2022. All patients diagnosed with TGA in their infancy were approached to participate. Children with double-outlet right ventricle and outflow tract or arch obstruction were excluded from the analysis. Patients with spontaneous genetic disorders, congenital anomalies, severe intellectual disabilities, or severe nerve injury (e.g., bacterial meningitis or head trauma or disorders of the central nervous system) not related to open chest surgery were also excluded. Parents fill out the SNAP-IV questionnaire by an electronic questionnaire and a telephone interview. Pediatricians will further understand the general details and disease status of the child by telephone interview. The study was approved by the Ethics Review Committee of Shanghai Children’s Medical Center (number: SCMCIRB-K2022042-1). This study has been registered with the Chinese Clinical Trials Registry^1^ as ChiCTR2200062243.

### Questionnaires, Swanson, Nolan, and Pelham IV, and scoring

The children’s parents were asked to perform a series of tests, which included the Swanson, Nolan, and Pelham IV (SNAP-IV) questionnaire, which is designed to quantify ADHD symptoms as described by the criteria of the Diagnostic and Statistical Manual of Mental Disorders, Fifth Edition. This tool enables the separate measurement of inattention and hyperactivity (12). Briefly, the SNAP-IV includes 26 items, the first nine of which assess core symptoms of inattention according to the Diagnostic and Statistical Manual of Mental Disorders, Fifth Edition (DSM-V). Items 10–18 correspond to core ADHD symptoms in the DSM-V in relation to hyperactive impulses. The last items (12–19) correspond to the core symptom of opposition, which is not discussed in this article (20, 21), and basic information about their children in a General Personal Information Summary, which includes sex, age, birth weight, height, weight, age of pregnancy, gestational age, family economic parental education, outpatient review time and other neurological problems. The questionnaire aimed to provide general information about the children’s developmental history and it contained questions pertaining to basic personal information, as well as neurologic development services used by the child. Parents were also asked to report whether their child had difficulties in school, received remedial educational services, or was able to perform mathematical calculations well.

We analyzed the prevalence of ADHD-like symptoms based on the SNAP-IV results. This involved the quantification of positive symptom scores (2 or 3 scales) of the inattention and hyperactivity/impulsivity items. Each item was rated using a 4-point Likert scale, with 0 indicating “never,” 1 “occasional,” 2 “regular,” and 3 “always.” If participants reported more than six positive symptoms in any of the two subscales, they were diagnosed with ADHD-like symptoms of predominantly inattentive or hyperactive/impulsive subtypes. If a participant presented symptoms of both inattention and hyperactivity/impulsivity, a combined subtype of ADHD symptoms was diagnosed (12). The ADHD-like symptoms prevalence in our cohort was compared with those of normal age-matched populations worldwide (22, 23).

### Characteristic

The primary outcomes were high-risk scores for inattention and hyperactivity based on the parental answers in the SNAP-IV questionnaire. For the risk analysis of adverse primary outcomes, identified perioperative variables included sex, gestational age, low birth weight, preoperative oxygen saturation (SpO_2_), surgical age, surgical weight, surgical height, alprostadil use, DHCA (Deep Hypothermic Circulatory Arrest), CPB (cardiopulmonary bypass), total cardiac support time, delayed sternal closure, positive inotropic drug score, and length of stay.

### Statistical analyses

In the baseline data, categorical variables were analyzed using the chi-square and Fisher’s precision tests. The distributions of continuous variables were detected using histograms and QQ plots. Two-sample independent *t*-tests were used to analyze continuous data that followed the normal distribution. Continuous data with non-normal distributions were analyzed using the Mann–Whitney *U*-test. Risk factors for the development of ADHD-like symptom after TGA surgery were explored using logistic regression. The variables included in the multivariate analysis were selected before the univariate analysis based on clinical relevance. *P* < 0.05 was considered to be statistically significant. Results were expressed as odds ratios (ORs) with 95% confidence intervals (95% CIs). Statistical analyses were conducted using IBM SPSS Statistics for Windows, version 26.0 (IBM Corp., Armonk, NY, United States).

## Results

### Attention deficit hyperactivity disorder-like symptoms epidemiology in the Great Arteries-treated patients

**FIGURE 1:**
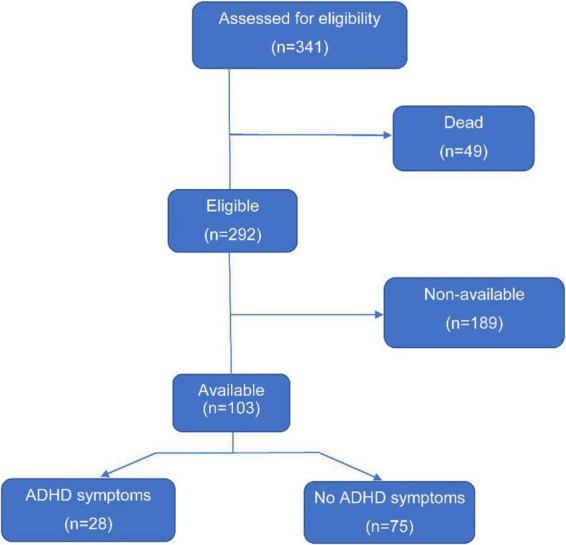
Patient selection flow chart.

The final analysis included 103 patients (83 boys and 20 girls) who were above 6 years (Figure 1). Among the 103 analyzed patients, 40 (38.8%) patients received preoperative mechanical ventilation, drugs were preoperatively administered to 58 (56.3%) children, and emergency surgery was performed in 33 (32.0%) patients before surgery. Postoperative peritoneal dialysis was performed in 22 (21.4%) patients with TGA. Basic demographic and clinical data are presented in Table 1.

**TABLE 1:** Demographic and clinical characteristics of 103 children with congenital heart disease.

Variable	*n* (%)
Sex (male)	83 (80.6)
Emergency surgery	33 (32)
Vascular malformation	20 (19.4)
**TGA subtype**	
VSD	60 (58.3)
IVS	43 (41.7)
**Cardiopulmonary bypass**	
Normal temperature	58 (56.3)
Not normal temperature	45 (43.7)
Alprostadil use	58 (56.3)
Breastfeeding	23 (22.3)
Preoperative ventilator use	40 (38.8)
Peritoneal dialysis	22 (21.4)
Postoperative rescuing	23 (22.3)
Second operation	8 (7.8)
**Mode of delivery**	
Natural birth	43 (41.7)
Cesarean delivery	60 (58.3)
**Parental education**	
Primary school	6 (5.8)
Junior high school	32 (31.1)
Senior high school	20 (19.4)
University	38 (36.9)
Postgraduate and above	7 (6.8)
**Household income (yuan/month)**	
≤5,000	36 (40.0)
>5,000	67 (65.0)
Length of hospital stay/day	19.2 ± 6.8 (8–45)
Surgical age/day	47.1 ± 70.2 (1–383)
Surgical weight/g	3908.5 ± 1209.7 (2100–8800)
Surgical height/cm	52.9 ± 5.6 (40–74)
Preoperative HCT	43.5 ± 7.3 (24.7–68)
Total time of cardiopulmonary bypass/min	137.9 ± 51.1(42–435)
Myocardial ischemia time/min	86.2 ± 26.3 (18–180)
CICU time/day	11.2 ± 5.9 (4–39)
Ventilator use/min	133.9 ± 112.5 (4–816.5)
Preoperative SpO_2_	68.7 ± 15.0 (21–95)
Time of chest catheter placement/day	7.9 ± 2.8 (3–17)
Delayed sternal closure/day	1.4 ± 1.5 (0–5)
Positive inotropic drug score/μg/kg/min	17.0 ± 7.0 (7–47.5)
Birth weight/g	3183.2 ± 503.5 (1900–4550)

CICU, cardiac surgery intensive care unit; DHCA, deep hypothermic circulatory arrest; HCT, hematocrit; IVS, intact ventricular septum; SpO_2_, oxygen saturation; TGA, transposition of the great arteries; VSD, ventricular septal defect; SD, Standard Deviation, IQR, Interquartile Range.

Of the patients who had delayed sternal closure, 21 patients developed ADHD-like symptoms, accounting for 38.2% of those patients. By contrast, among the patients without delayed sternal closure, only 7 (14.6%) children developed ADHD-like symptoms (OR, 3.62; 95% CI: 1.37–9.53; chi-square test; Table 2). The mean preoperative SpO_2_ in patients with ADHD-like symptoms was 62.54% compared with 71.17% in patients without ADHD-like symptoms (95% CI: 2.07–14.86). The medium surgical weight at the time of surgery in patients with TGA and ADHD-like symptoms was 3,500 g vs. 3,800 g in patients without ADHD-like symptoms (95% CI: 0.009–0.013). Likewise, the medium surgical height was 50 cm in TGA patients with ADHD-like symptoms and 51 cm in those without ADHD-like symptoms (95% CI, 0.01–0.02).

**TABLE 2:** Clinical variables for patients with and without ADHD-like symptoms in D-TGA-treated patients.

Characteristics number, *n*	ADHD-like symptoms (*n* = 28)	Non-ADHD like symptoms (*n* = 75)	*P*
Sex, *n* (%)			0.60
Female	4 (3.9%)	16 (15.5%)	
Male	24 (23.3%)	59 (57.3%)	
Emergency surgery, *n* (%)	12 (11.7%)	21 (20.4%)	0.15
Vascular malformation, *n* (%)	5 (4.9%)	15 (14.6%)	0.81
TGA subtype, *n* (%)			0.02
IVS	17 (16.5%)	26 (25.2%)	
VSD	11 (10.7%)	49 (47.6%)	
Cardiopulmonary bypass, *n* (%)			0.73
Normal temperature	15 (14.6%)	43 (41.7%)	
Not Normal temperature	13 (12.6%)	32 (31.1%)	
Delayed sternal closure, *n* (%)	21 (20.4%)	34 (33%)	0.01
Alprostadil use, *n* (%)	18 (17.5%)	40 (38.8%)	0.32
Breastfeeding, *n* (%)	4 (3.9%)	19 (18.4%)	0.35
Preoperative ventilator use, *n* (%)	12 (11.7%)	28 (27.2%)	0.61
Peritoneal dialysis, *n* (%)	9 (8.7%)	13 (12.6%)	0.10
Postoperative rescuing, *n* (%)	6 (5.8%)	17 (16.5%)	0.89
Second operation, *n* (%)	1 (1%)	7 (6.8%)	0.58
Mode of delivery, *n* (%)			0.76
Natural birth	11 (10.7%)	32 (31.1%)	
Cesarean delivery	17 (16.5%)	43 (41.7%)	
Parental education, *n* (%)			0.57
Primary school	2 (1.9%)	4 (3.9%)	
Junior high school	8 (7.8%)	24 (23.3%)	
Senior high school	3 (2.9%)	17 (16.5%)	
University	12 (11.7%)	26 (25.2%)	
Postgraduate and above	3 (2.9%)	4 (3.9%)	
Length of hospital stay, median (IQR)	18 (15, 23)	18 (14, 23)	0.72
Surgical age, median (IQR)	11.5 (8, 41)	25 (9, 60)	0.20
Surgical weight, median (IQR)	3500 (2900, 3875)	3800 (3300, 4400)	0.01
Surgical height, median (IQR)	50 (50, 51.75)	51 (50, 56)	0.02
Preoperative HTC, mean ± SD	42.51 ± 5.65	43.82 ± 7.78	0.42
Total time of cardiopulmonary bypass, median (IQR)	114 (101, 136)	135 (115, 152)	0.05
Myocardial ischemia time, median (IQR)	75 (63, 94)	85 (73, 96)	0.06
CICU time, median (IQR)	10 (8, 16)	8 (7, 14)	0.04
Ventilator use, median (IQR)	131.75 (94.58, 162.75)	93.9 (69, 161.5)	0.04
Preoperative SpO_2_, mean ± SD	62.54 ± 18.07	71.00 ± 13.05	0.01
Time of chest catheter placement, median (IQR)	7 (6, 10.75)	8 (6, 9)	0.83
Positive inotropic drug score, median (IQR)	17.55 (13.73, 22.5)	14.5 (11.8, 19.5)	0.01

CICU, cardiac surgery intensive care unit; DHCA, deep hypothermic circulatory arrest; HCT, hematocrit; IQR, interquartile range; IVS, intact ventricular septum; SpO_2_, oxygen saturation; TGA, transposition of the great arteries; VSD, ventricular septal defect.

After surgery, the medium days in the Cardiac Intensive Care Unit for patients with ADHD-like symptoms were 10 days vs. 8 days in patients with TGA and without ADHD -like symptoms (95% CI, 0.01–0.02). Moreover, postoperative ventilator use was more common in ADHD-like symptoms patients. The interquartile range of positive inotropic drug score was 17.55 μg/kg/min in patients with ADHD-like symptoms vs. 14.5 μg/kg/min than in those without (95% CI, 0.03–0.04). Among the parameters with significant group differences were also the total time of extracorporeal circulation and myocardial ischemia time. A comparison of the clinical variables for patients with and without ADHD-like symptoms is provided in Table 2.

**FIGURE 2:**
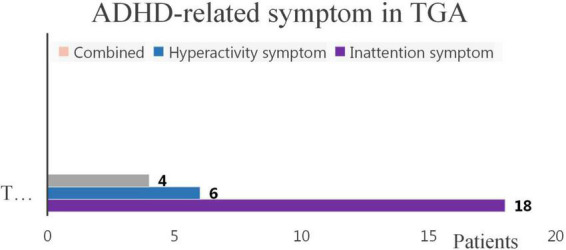
Inattention, hyperactivity, and combined symptoms in TGA-treated patients. *ADHD*, attention-deficit/hyperactivity disorder; *TGA*, transposition of the great arteries. This chart illustrates that in TGA patients, there were a total of 28 patients with ADHD-like symptoms, including 18 patients with inattention symptoms, 6 hyperactivity patients and 4 patients with combined symptoms.

The prevalence of ADHD-like symptoms in patients after TGA surgery was 27.18%. Inattention, hyperactivity/impulsivity, and combined symptoms were present in 17.5, 5.8, and 3.8% of TGA patients, respectively (Figure 2). The percentage patients with ADHD-like symptoms presented as inattention-predominant subtypes, but not of combined or hyperactivity/impulsivity-predominant subtypes, was significantly higher in TGA patients. The prevalence of inattention-related symptoms in our study population was significantly higher than those in age-matched normal populations worldwide.

### Risk factors of attention deficit hyperactivity disorder-like symptoms

We analyzed possible risk factors for the development of ADHD-like symptoms after TGA surgery (Table 3). Univariate analysis showed that positive inotropic drug score (OR, 1.07; 95% CI, 1.00–1.14) and delayed sternal closure (OR, 1.46; 95% CI, 1.07–1.97) were risk factors of ADHD-like symptoms, while increased preoperative SpO_2_ (OR, 0.96; 95% CI, 0.94–0.99), surgical height (OR, 0.86; 95% CI, 0.76–0.97) and TGA subtype (VSD) (OR, 0.34; 95% CI, 0.14–0.84) were protective factors. After multivariate regression analysis, only delayed sternal closure (OR, 1.49; 95% CI, 1.02–2.18) remained independent risk factors and preoperative increased SpO_2_ (*P* < 0.01; OR, 0.95; 95% CI, 0.92–0.99) is a protective factor (Table 3). Using the same parameters for the patients with TGA, we also analyzed the risk factors for inattention- and hyperactivity/impulsivity-related symptoms. Multivariate regression analysis indicated that delayed sternal closure (OR, 1.81; 95% CI, 1.07–3.06) and positive inotropic drug use (OR, 1.10; 95% CI, 1.00–1.20) were significant risk predictors of inattention-related symptoms and increased preoperative SpO_2_ (*P* < 0.01, OR, 0.91; 95% CI, 0.87–0.96) is a protective factor (Table 4).

**TABLE 3:** Risk analysis of perioperative variables for ADHD-like symptoms.

Variable/ADHD-like symptoms risk	Univariate factor			Multivariate factor		
	OR	95% CI	*P*	OR	95% CI	*P*
Sex (male or female)	1.63	0.49–5.37	0.42			
Birth weight/g	1	1.00–1.001	0.88			
**Household income/yuan per monthly**						
<5,000		[Reference]				
>5,000	0.77	0.32–1.90	0.57			
Parental education			0.59			
Primary school		[Reference]				
Junior high school	0.67	0.10–4.35	0.67			
Senior high school	0.35	0.04–2.87	0.33			
University	0.92	0.15–5.75	0.93			
Above all	1.50	0.16–14.42	0.73			
Breastfeeding	2.04	0.63–6.62	0.24			
Surgical weight/g	1	0.99–1	0.07			
Surgical height/cm	0.86	0.76–0.97	0.01*			
Preoperative SpO_2_	0.96	0.94–0.99	0.01*	0.95	0.92–0.99	0.006*
Preoperative HCT	0.97	0.92–1.04	0.41			
Alprostadil use	1.58	0.64–3.86	0.32			
Preoperative ventilator use	1.26	0.52–3.04	0.61			
Surgical age/day	0.99	0.98–1.00	0.14			
Emergency surgery	1.93	0.78–4.76	0.15			
Vascular malformation	0.81	0.28–2.67	0.81			
**TGA subtype**						
IVS		[Reference]				
VSD	0.34	0.14–0.84	0.02*			
Cardiopulmonary bypass						
Normal temperature		[Reference]				
Not normal temperature	1.17	0.49–2.79	0.73			
Preoperative ventilator use	1.26	0.52–3.04	0.61			
Delayed sternal closure/day	1.46	1.07–1.97	0.02*	1.49	1.02–2.18	0.037*
Cardiopulmonary bypass/min	0.99	0.98–1.00	0.10			
Myocardial ischemia time/min	0.98	0.96–1.00	0.07			
Postoperative SpO_2_	1.01	0.98–1.05	0.45			
Postoperative rescuing	0.93	0.33–2.67	0.90			
Positive inotropic drug score	1.07	1.00–1.14	0.03*			
CICU time/day	1.06	0.98–1.13	0.13			
Peritoneal dialysis	2.26	0.84–6.10	0.11			
Chest catheter placement/day	1.03	0.88–1.20	0.72			
Length of hospital stay/day	1.01	0.95–1.07	0.80			
Second operation	0.36	0.04–3.07	0.35			
Mode of delivery	1.15	0.47–2.79	0.76			

ADHD, attention-deficit/hyperactivity disorder; CI, confidence interval; DHCA, deep hypothermic circulatory arrest; HCT, hematocrit; IQR, interquartile range; OR, dominance ratio; SpO_2_, oxygen saturation; TGA, transposition of the great arteries.

*Only significant variables provided.

**TABLE 4:** Risk analysis of perioperative variables for inattention-related symptoms.

	Univariate factor			Multivariate factor		
	OR	95% CI	*P*	OR	95% CI	*P*
Sex (male or female)	1.25	0.32–4.82	0.75			
Birth weight/g	1.00	0.99–1.00	0.99			
Household income/yuan per month			0.56			
<5,000		[Reference]				
>5,000	0.81	0.29–2.32	0.70			
Parental education			0.87			
Primary school		[Reference]				
Junior high school	1.40	0.14–14.03	0.78			
Senior high school	0.56	0.04–7.46	0.66			
University	1.13	0.11–11.24	0.92			
Above all	0.83	0.04–16.99	0.91			
Breastfeeding	5.94	0.75–47.25	0.09			
Surgical weight/g	1.00	0.99–1.00	0.23			
Surgical height/cm	0.86	0.75–0.99	0.04			
Preoperative SpO_2_	0.95	0.91–0.98	0.00	0.91	0.87–0.96	0.001*
Preoperative HCT	0.99	0.93–1.07	0.91			
Alprostadil use	2.31	0.76–7.05	0.14			
Preoperative ventilator use	1.74	0.63–4.85	0.29			
Surgical age/day	0.99	0.98–1.01	0.23			
Emergency surgery	3.37	1.18–9.59	0.02			
Vascular malformation	0.80	0.21–3.08	0.75			
**TGA subtype**						
IVS		[Reference]				
VSD	0.38	0.14–1.09	0.07			
**Cardiopulmonary bypass**						
Normal temperature		[Reference]				
Not Normal temperature	0.43	0.14–1.32	0.14			
Preoperative ventilator use	1.74	0.63–4.85	0.29			
Delayed sternal closure/day	1.55	1.08–2.22	0.02	1.807	1.07–3.06	0.03*
Cardiopulmonary bypass/min	0.99	0.97–1.00	0.11			
Myocardial ischemia time/min	0.98	0.96–1.00	0.17			
Postoperative rescuing	0.99	0.29–3.37	0.99			
Positive inotropic drug score	1.10	1.03–1.18	0.00	1.096	1.01–1.20	0.04*
CICU time/day	1.07	0.99–1.15	0.09			
Peritoneal dialysis	2.97	0.99–8.92	0.05			
Chest catheter placement/day	0.94	0.77–1.15	0.55			
Length of hospital stay/day	0.99	0.92–1.07	0.88			
Second operation	0	0	0.99			
Mode of delivery	0.88	0.31–2.44	0.80			

ADHD, attention-deficit/hyperactivity disorder; CI, confidence interval; DHCA, Deep Hypothermic Circulatory Arrest; HTC, Hematocrit; IQR, interquartile range; OR, dominance ratio; SpO_2_, oxygen saturation; TGA, transposition of the great arteries.

*Only significant variables provided.

### Academic and educational outcomes

Based on the follow-up information provided by parents, 67.8% with ADHD-like symptoms ranked below average or well below average in patients with TGA. In contrast, there was 64% ranked above average or well above average without ADHD-like symptoms.

**FIGURE 3:**
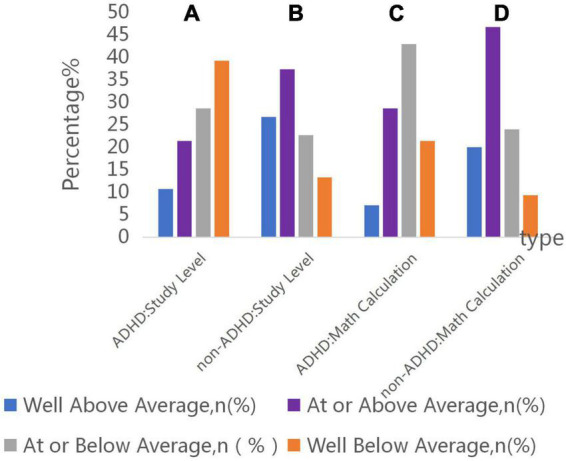
Parent rating of child’s academic and mathematical performance. **(A)** Number of these patients with ADHD-like symptoms of study level in well below average, at or below average, at or above average, well above average was 11 (39.2%), 8 (28.6%), 6 (21.4%), 3 (10.7%). **(B)** Number of these patients without ADHD-like symptoms of study level in well below average, at or below average, at or above average, well above average was 10 (13.3%), 17 (22.7%), 28 (37.3%), 20 (26.7%). **(C)** Number of these patients with ADHD-like symptoms of mathematical level in well below average, at or below average, at or above average, well above average was 6 (21.4%), 12 (42.9%), 8 (28.6%), 2 (7.1%). **(D)** Number of these patients without ADHD-like symptoms of mathematical level in well below average, at or below average, at or above average, well above average was 7 (9.3%), 18 (24%), 35 (46.7%), 15 (20%).

In terms of math scores, there is only 7.1% of children with ADHD-like symptoms ranked in upper level, whereas in children without ADHD-like symptoms, 20% math scores were in upper level (Figure 3).

**FIGURE 4:**
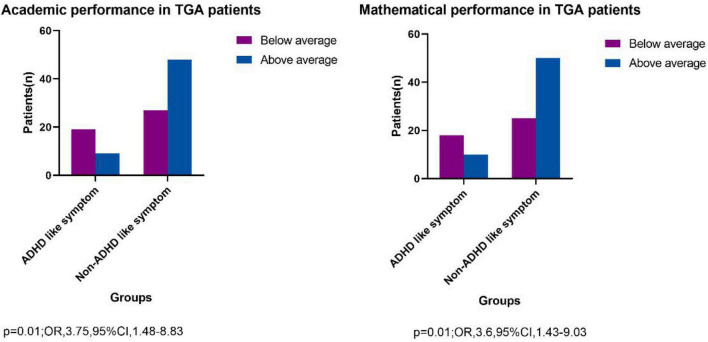
Academic and mathematical performance in TGA patients.

There was statistical difference between them (*P* = 0.01; OR, 3.75; 95% CI, 1.48–8.83) in below average study performance and above average performance in group of ADHD-like symptoms and Non-ADHD like symptoms. Besides, the ADHD-like symptoms group had a higher percentage of mathematics performance below average, there was statistical difference between the two groups (*P* = 0.01; OR, 3.6; 95% CI, 1.43–9.03) (Figure 4).

We also counted the percentage of children who had completed surgery with well below average academic performance in study level is 20.4% and in mathematical level is 12.6%.

### Special education-seeking behavior

**FIGURE 5:**
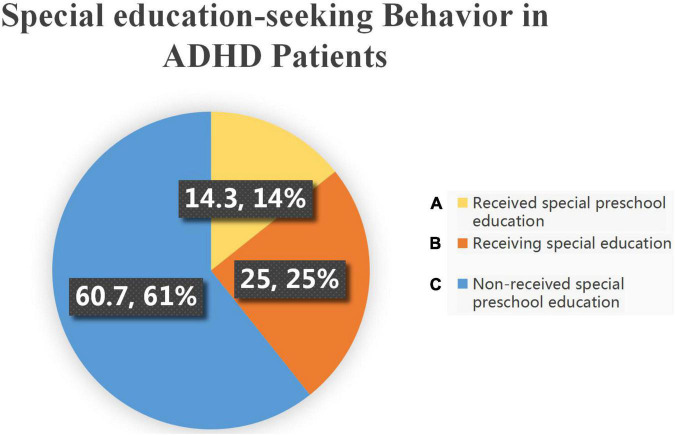
Distribution of special preschool education services used by children in this cohort. **(A)** Of these TGA patients with ADHD-like symptoms, there were 4 (14.3%) patients who had received special preschool education, **(B)** 7 (25%) patients were receiving special education, and **(C)** 17 (60.7%) patients with no special education received.

According to our research, only 14.3% of these 28 probable patients had ever sought help in special preschool education. Furthermore, 60.7% of the parents of CHD children with ADHD-like symptoms did not notice considerable problems. In addition, only 25% of these patients were receiving special education (Figure 5). In the subtype analysis, parents of children with isolated inattention-related symptoms had the lowest help-seeking rate. The main cause for this result was the parents’ belief that treatment for TGA was more important. This implies that knowledge about ADHD-like symptoms among parents of children with CHD is low and that they may underestimate the crucial role of early ADHD-like symptoms intervention.

## Discussion

In this study we found 27.2% of a cohort of patients with D-TGA after ASO presented ADHD-like symptoms. Clinical multivariable analysis showed that decreased preoperative SpO_2_ and postoperative DSC are two independent risk factors for ADHD-like symptoms.

### Attention deficit hyperactivity disorder-like symptoms profiles in patients after arterial switch operation

The high occurrence of ADHD symptoms have been described in children with variety of CHD. Mahle et al. reported 69.5% of children with hypoplastic left heart syndrome had the symptoms of inattention, hyperactivity, or both (24). Kirshbom et al. reported that 50% of children with total anomalous pulmonary venous return displayed abnormal hyperactivity, attention deficits, or both (25). Hövels-Gürich et al. found that the tetralogy of Fallot group demonstrated poor attention skills as compared with the ventricular septal defect group and the healthy control subjects (26). Hansen et al. also found that ADHD symptoms are more prevalent in children with cyanotic CHD than acynotic CHD (9). Shillingford et al. identified that the number of children with complex CHD receiving clinically significant scores for inattention and hyperactivity was 3–4 times higher than observed in the general population (11). Moreover, recent studies showed that patients with a simple CHD also have a higher ADHD symptom burden compared with population means and controls in adults (27, 28). Therefore, ADHD-like symptoms has now become a common neurobehavioral and psychiatric disorder after congenital heart surgery.

D-TGA is one of the most extensively studied cyanotic CHD with regard to neurodevelopmental outcomes. Long-term follow-up of these children who underwent the ASO reveals multiple neurologic deficits in cognitive function, motor skills, academic achievement, and language ability in infancy and early childhood, and as they were followed into their teenage years, behavioral problems and subsequently psychiatric problems became increasingly prominent (29, 30). Bellinger et al. found that their patients had difficulty with higher-order executive function, including sustained attention 8 years after ASO (31). DeMaso et al. found that the most common psychiatric diagnosis in adolescent with D-TGA was ADHD symptoms, present in 16% of subjects (32). Holst et al. also showed that inattention symptom loads were in 15.2% of the children with D-TGA (33). Surprisingly, in the present study, the prevalence of ADHD-like symptom was up to 27.2% in similar D-TGA population, though using the same diagnostic criteria. The reasons for this significant differences are not clear, but we speculated that it is at least partly related to our suboptimal perioperative management strategy including low prenatal diagnosis rates, late referral due to the parents’ knowledge of this lesion or lack of effective transport mechanisms, which increase the time of cyanosis and pulmonary over-circulation, may contribute to the impaired growth of the brain and thereby delayed neurodevelopment (13).

In our study, it was found that inattention, hyperactivity and the combination of the two accounted for about 64.3, 28.6, and 14.3% of ADHD-like symptoms, respectively. Consistent with other studies, inattention was the main symptom. Less than 15% of those children with ADHD-like symptoms requiring special education received additional education.

Our findings are different from those of the Boston Circulatory Arrest Study, which prospectively followed a cohort of infants with dextrotransposition of TGA. Different to its report, one-third of the children were receiving school support, and 10% of the children had already repeated a grade, our research found that twenty five percent received remedial education, and none had repeated grades.

Mahle and Wernovsky (6) reported the school-age follow-up in a group of children with hypoplastic left heart syndrome and found that 34% were receiving special services in school. However, in our research, we could find there are indeed some patients receive remedial special education support.

### Potential mechanisms of attention deficit hyperactivity disorder-like symptoms in patients after arterial switch operation

The mechanisms for neurodevelopmental impairment in CHD are multifactorial. Early investigators attributed neurologic injury to a direct consequence of surgical repair and particularly exposure to cardiopulmonary bypass or circulatory (14), and later, genetic syndrome, preoperative epilepsy, various complications, timing of mechanical ventilation, and longer postoperative hospital stays and socioeconomic disadvantage had been reported as risk factors for neurodevelopment abnormalities in patients with CHD (15, 16). In adolescents with D-TGA, impaired cognitive functioning and parental stress at younger age emerged as significant risk factors for increased rates of ADHD symptoms (32). However, all the above clinical covariates were included and analyzed in this study, and it was not found that these factors were correlated to the occurrence of ADHD-like symptoms, but we confirmed that preoperative hypoxia was related to the occurrence of ADHD-like symptoms like other studies (9, 11, 26).

In fetus and unrepaired neonates with D-TGA, oxygen supply to brain tissue reduces due to abnormal ventricular-arterial connection and abnormal hemodynamics. Chronic hypoxia may cause brain developmental immaturity and cerebral white matter injury seen on MRI neuroimaging, which are related to behavioral changes in children with CHD (4). Moreover, acute hypoxemia can cause injury to the prefrontal cortex and corpus striatum of the brain (26). The striatum, located at the border of the cerebral artery supply territories, represents a watershed area between deep penetrating and superficial blood vessels. Therefore, the striatum and thalamus are extremely susceptible to changes in the circulatory system. As the striatum is also rich in spiny neurons, it aggregates almost the entire neuronal activity of the cortex. Thus, the striatum is stimulated by hypoxia or ischemic conditions to release excessive amounts of neurotransmitters and have been associated with shaping response to emotion, inhibition of socially unacceptable behavior and impaired executive control network of attention (17).

Interestingly, DSC was also identified to be an independent risk factor for ADHD-like symptoms occurrence in this study. As a management strategy for hemodynamic instability and severe coagulopathy after ASO, DSC could decrease the early surgical mortality and morbidity of patients with D-TGA (18), though no previous studies have found DSC to be associated with neurodevelopment abnormality of CHD. We speculated that DSC may led to longer ICU stays and hospital stays and an increase in the rate of hospital infection, which may comprehensively affect neurodevelopmental consequences. On the other hand, unstable cardiac function in patients with DSC might also impair blood supply to ADHD-like symptoms associated sensitive brain domain in a way. Increased inotropic score was a risk factor in univariate analysis for the attention symptom in this study, which also demonstrated that hemodynamic instability is indeed a non-negligible factor for ADHD-like symptoms occurrence.

So we also could take some measures to help these patients reduce the risk of ADHD-like symptoms. First of all, using some appropriate vasoconstriction and enhanced blood pressure drugs for some preoperative children. Appropriate blood transfusion can be used to increase ventilation and improve the oxygen carrying capacity of red blood cells. However, due to the defection of the current research mechanism, this method should be used with caution in combination of childrens’ condition.

Secondly, the protection of myocardium can be strengthened by selecting myocardial protective fluid during surgery.

Thirdly, after the operation, we should strengthen the caring for the serious degree of CHD and use timely anticoagulant drug to reduce the duration of infection risk CICU.

### Clinical implications and future direction

Patients with D-TGA undergoing ASO, constitute a group receiving a homogeneous treatment regimen. This facilitates the assessment of the relative contributions of preoperative, intraoperative and postoperative risk factors to neurological outcomes. The main finding in our study is that the prevalence of ADHD-like symptoms was significantly higher than that in Western countries (32, 33). Meanwhile, half of the parents are unaware of ADHD-like symptoms and its harmfulness, only about one-fifth of parents sought special treatment, in particular, parents of children with isolated inattention symptoms were less likely to seek special help. Therefore, primary care clinicians and cardiac team should be alert to the early identification of ADHD-like symptoms in the children and adolescents with D-TGA after ASO during the follow-up, and provide them with appropriate medical treatments or interventions to improve their quality of life (19, 27).

In this study, preoperative decreased hypoxia and DSC were identified to be two potentially modifiable risk factors for ADHD-like symptoms, which imply that ASO should be performed as soon as possible to shorten the chronic hypoxic time from birth to surgery and reduce the risk of brain injury from preoperative acute hypoxic events (34, 35), and some surgical maneuvers also need to be performed to reduce surgical trauma, duration of cardiopulmonary bypass and cross clamp time to decrease the risk of DSC. The causal relationship between hypoxia and ADHD-like symptoms will be studied in future to establish the associate pathogenesis of ADHD, and provide practical and clinically significant options for the prevention and treatment of ADHD after ASO.

### Limitations

Our study has some limitations. First, the screen of ADHD was based on SNAP-IV. However, these criteria are highly sensitivity, lacking of some specialty (36), so we still needed to be cautious in determining and interpreting the results in this study. Second, we only preliminarily investigated the prevalence and risk factors of ADHD-like symptom. We did not set the normal population or other simple CHD as the control group, so we could not effectively compare the prevalence of ADHD-like symptoms in children with various CHD. Meanwhile, we did not evaluate other neurodevelopmental disorders and could not fully reflect the overall neurodevelopmental status of children in patients with D-TGA after ASO and its relationship with ADHD-like symptoms. Third, this cohort study lacked neuroimaging studies and failed to evaluate the relationship between the brain structural and functional abnormalities and ADHD-like symptoms. Fourth, some of the conclusions were speculative and needed further validation.

## Conclusion

In this study we concluded that children and adolescents with D-TGA undergoing ASO are at high risk of ADHD-like symptoms, especially inattention-related symptoms. Preoperative hypoxia and postoperative DSC are two high-risk factors. It is vital to carry out further research and strengthen our evaluation, with regard to surveillance and management of neurodevelopmental function, especially ADHD-like symptoms in children and adolescents with D-TGA after ASO.

## Data availability statement

The raw data supporting the conclusions of this article will be made available by the authors, without undue reservation.

## Ethics statement

Written informed consent was not obtained from the minor(s)’ legal guardian/next of kin for the publication of any potentially identifiable images or data included in this article.

## Author contributions

HTC: writing and designing original draft. YY: formal analysis. CL and XZ: methodology. GW: validation of research. ZJ: conducting a research. GS and XH: investigation. XT and HWC: supervision. ZZ: conceptualization and revise original draft. All authors contributed to the article and approved the submitted version.

## Footnotes

1
https://www.chictr.org.cn

## References

[1] Fulton DR Fyler DC. D-transposition of the great arteries. 2nd ed. In: Keane JF Lock JE Fyler DC editors. *Nadas’ Pediatric Cardiology.* Amsterdam: Amsterdam (2006). p. 645.

[2] Hutter PA Kreb DL Mantel SF Hitchcock JF Meijboom EJ Bennink GB. Twenty-five years’ experience with the arterial switch operation. *J Thorac Cardiovasc Surg.* (2002) 124:790. 12324738 10.1067/mtc.2002.120714

[3] Rudra HS Mavroudis C Backer CL Kaushal S Russell H Steward RD The arterial switch operation: 25-year experience with 258 patients. *Ann Thorac Surg.* (2011) 92:1742–6. 10.1016/j.athoracsur.2011.04.101 21925641

[4] Bellinger DC Wypij D Rivkin MJ DeMaso DR Robertson RL Jr. Dunbar-Masterson C. Adolescents with d-transposition of the great arteries corrected with the arterial switch procedure: neuropsychological assessment and structural brain imaging. *Circulation.* (2011) 124:1361–9. 10.1161/circulationaha.111.026963 21875911 PMC3217719

[5] Andropoulos DB Easley RB Brady K McKenzie ED Heinle JS Dickerson HA Changing expectations for neurological outcomes after the neonatal arterial switch operation. *Ann Thorac Surg.* (2012) 94:1250–6. 10.1016/j.athoracsur.2012.04.050 22748448 PMC3586524

[6] Mahle WT Wernovsky G. Neurodevelopmental outcomes in hypoplastic left heart syndrome. *Semin Thorac Cardiovasc Surg Pediatr Card Surg Annu.* (2004) 7:39–47. 15283351 10.1053/j.pcsu.2004.02.019

[7] Spencer TJ Biederman J Mick E. Attention-deficit/hyperactivity disorder:diagnosis, lifespan, comorbidities, and neurobiology. *J Pediatr Psychol.* (2007) 32:631–42. 10.1093/jpepsy/jsm005 17556405

[8] Katzman MA Bilkey TS Chokka PR Fallu A Klassen LJ. Adult ADHD and comorbid disorders:clinical implications of a dimensional approach. *BMC Psychiatry.* (2017) 17:302–16. 10.1186/s12888-017-1463-3 28830387 PMC5567978

[9] Hansen E Poole TA Nguyen V Lerner M Wigal T Shannon K Prevalence of ADHD symptoms in patients with congenital heart disease. *Pediatr Int.* (2012) 54:838–43. 10.1111/j.1442-200x.2012.03711.x 22882233

[10] Wang CC Weng WC Chang LY Chang HY Wu MH Wang JK Increased prevalence of inattention-related symptoms in a large cohort of patients with congenital heart disease. *Eur Child Adolesc Psychiatry.* (2021) 30:647–55. 10.1007/s00787-020-01547-y 32394091

[11] Shillingford AJ Glanzman MM Ittenbach RF Clancy RR Gaynor JW Wernovsky G. Inattention, hyperactivity, and school performance in a population of school-age children with complex congenital heart disease. *Pediatrics.* (2008) 121:e759–67. 10.1542/peds.2007-1066 18381503

[12] Liu YC Liu SK Shang CY Lin CH Tu C Gau SS. Norm of the Chinese version of the Swanson, Nolan and Pelham, version IV scale for ADHD. *Taiwan J Psychiatry.* (2006) 20:290–304. 10.1002/mpr.237 18286459 PMC6878250

[13] Peyvandi S De Santiago V Chakkarapani E Chau V Campbell A Poskitt KJ Association of prenatal diagnosis of critical congenital heart disease with postnatal brain development and the risk of brain injury. *JAMA Pediatr.* (2016) 170:e154450. 10.1001/jamapediatrics.2015.4450 26902528 PMC5083633

[14] Hsia TY Gruber PJ. Factors influencing neurologic outcome after neonatal cardiopulmonary bypass: what we can and cannot control. *Ann Thorac Surg.* (2006) 81:S2381–8. 10.1016/j.athoracsur.2006.02.074 16731107

[15] Howell HB Zaccario M Kazmi SH Desai P Sklamberg FE Mally P. Neurodevelopmental outcomes of children with congenital heart disease: a review. *Curr Probl Pediatr Adolesc Health Care.* (2019) 49:100685. 10.1016/j.cppeds.2019.100685 31708366

[16] Ryan KR Jones MB Allen KY Marino BS Casey F Wernovsky G Neurodevelopmental outcomes among children with congenital heart disease: at-risk populations and modifiable risk factors. *World J Pediatr Congenit Heart Surg.* (2019) 10:750–8. 10.1177/215035119878702 31658880

[17] Decker MJ Jones KA Solomon IG Keating GL Rye DB. Reduced extracellular dopamine and increased responsiveness to novelty: neurochemical and behavioral sequelae of intermittent hypoxia. *Sleep.* (2005) 28:169–76. 10.1093/sleep/28.2.169 16171240

[18] Nelson-McMillan K Hornik CP He X Vricella LA Jacobs JP Hill KD Delayed sternal closure in infant heart surgery—the importance of where and when: an analysis of the STS congenital heart surgery database. *Ann Thorac Surg.* (2016) 102:1565–72. 10.1016/j.athoracsur.2016.08.081 27720371

[19] Batra AS Alexander ME Silka MJ. Attention-deficit/hyperactivity disorder, stimulant therapy, and the patient with congenital heart disease: evidence and reason. *Pediatr Cardiol.* (2012) 33:394–401. 10.1007/s00246-012-0162-6 22298228

[20] Ayano G Yohannes K Abraha M. Epidemiology of attention-deficit/hyperactivity disorder (ADHD) in children and adolescents in Africa: a systematic review and meta-analysis. *Ann Gen Psychiatry.* (2020) 19:21. 10.1186/s12991-020-00271-w 32190100 PMC7071561

[21] Chen YL Chen SH Gau SS. ADHD and autistic traits, family function, parenting style, and social adjustment for Internet addiction among children and adolescents in Taiwan: a longitudinal study. *Res Dev Disabil.* (2015) 39:20–31. 10.1016/j.ridd.2014.12.025 25617844

[22] Gau SS Shang CY Liu SK Lin CH Swanson JM Liu YC Psychometric properties of the Chinese version of the Swanson, Nolan, and Pelham, version IV scale—parent form. *Int J Methods Psychiatr Res.* (2008) 17:35–44. 10.1002/mpr.237 18286459 PMC6878250

[23] Swanson JM Kraemer HC Hinshaw SP Arnold LE Conners CK Abikoff HB Clinical relevance of the primary findings of the MTA: success rates based on severity of ADHD and ODD symptoms at the end of treatment. *J Am Acad Child Adolesc Psychiatry.* (2001) 40:168–79. 10.1097/00004583-200102000-00011 11211365

[24] Mahle WT Clancy RR Moss EM Gerdes M Jobes DR Wernovsky G. Neurodevelopmental outcome and lifestyle assessment in school-aged and adolescent children with hypoplastic left heart syndrome. *Pediatrics.* (2000) 105:1082–9. 10.1542/peds.105.5.1082 10790466

[25] Kirshbom PM Flynn TB Clancy RR Ittenbach RF Hartman DM Paridon SM Late neurodevelopmental outcomes after repair of total anomalous pulmonary venous connections. *J Thorac Cardiovasc Surg.* (2005) 129:1091–6. 10.1016/j.jtcvs.2004.08.013 15867785

[26] Hövels-Gürich HH Konrad K Skorzenski D Herpertz-Dahlmann B Messmer BJ Seghaye MC. Attentional dysfunction in children after corrective cardiac surgery in infancy. *Ann Thorac Surg.* (2007) 83:1425–30. 10.1016/j.athoracsur.2006.10.069 17383351

[27] Lau-Jensen SH Asschenfeldt B Evald L Hjortdal VE. Hyperactivity and inattention in young patients born with an atrial septal or ventricular septal defect. *Front Pediatr.* (2021) 9:786638. 10.3389/fped.2021.786638 34938699 PMC8686760

[28] Asschenfeldt B Evald L Heiberg J Salvig C Østergaard L Dalby RB Neuropsychological status and structural brain imaging in adults with simple congenital heart defects closed in childhood. *J Am Heart Assoc.* (2020) 9:e015843. 10.1161/jaha.120.015843 32427039 PMC7428999

[29] Neufeld RE Clark BG Robertson CM Moddemann DM Dinu IA Joffe AR. Five-year neurocognitive and health outcomes after the neonatal arterial switch operation. *J Thorac Cardiovasc Surg.* (2008) 136:1413–21. 10.1016/j.jtcvs.2008.05.011 19114183

[30] Robson VK Stopp C Wypij D Dunbar-Masterson C Bellinger DC DeMaso DR Longitudinal associations between neurodevelopment and psychosocial health status in patients with repaired d-transposition of the great arteries. *J. Pediatr.* (2019) 204:38–45. 10.1016/j.jpeds.2018.08.069 30274922 PMC6309657

[31] Bellinger DC Wypij D Rappaport LA Jonas RA Wernovsky G Newburger JW. Neurodevelopmental status at eight years in children with dextrotransposition of the great arteries: the Boston Circulatory Arrest Trial. *J Thorac Cardiovasc Surg.* (2003) 126:1385–96. 10.1016/s0022-5223(03)00711-6 14666010

[32] DeMaso DR Labella M Taylor GA Forbes PW Stopp C Bellinger D. Psychiatric Disorders and Function in Adolescents with d-Transposition of the Great Arteries. *J Pediatr.* (2014) 165:760–6. 10.1016/j.jpeds.2014.06.029 25063716 PMC4177329

[33] Holst LM Kronborg JB Jepsen JR Christensen JØ Vejlstrup NG Juul K. Attention-deficit/hyperactivity disorder symptoms in children with surgically corrected Ventricular Septal Defect, Transposition of the Great Arteries, and Tetralogy of Fallot. *Cardiol Young.* (2020) 30:180–7. 10.1017/s1047951119003184 31928549

[34] Lynch JM Ko T Busch DR Newland JJ Winters ME Mensah-Brown K Preoperative cerebral hemodynamics from birth to surgery in neonates with critical congenital heart disease. *J Thorac Cardiovasc Surg.* (2018) 156:1657–64. 10.1016/j.jtcvs.2018.04.098 29859676 PMC6166233

[35] Petit CJ Rome JJ Wernovsky G Mason SE Shera DM Nicolson SC Preoperative brain injury in transposition of the great arteries is associated with oxygenation and time to surgery, not balloon atrial septostomy. *Circulation.* (2009) 119:709–16. 10.1161/circulationaha.107.760819 19171858 PMC2736632

[36] Austerman J. ADHD and behavioral disorders: assessment, management, and an update from DSM-5. *Cleve Clin J Med.* (2015) 82:S2–7. 10.3949/ccjm.82.s1.01 26555810

